# Commentary: The Dynamic Features of Lip Corners in Genuine and Posed Smiles

**DOI:** 10.3389/fpsyg.2018.01610

**Published:** 2018-09-25

**Authors:** Yingqi Li, Zhongyong Shi, Honglei Zhang, Lishu Luo, Guoxin Fan

**Affiliations:** ^1^School of Humanity, Tongji University, Shanghai, China; ^2^Psychiatry Department, Shanghai Tenth People's Hospital, Tongji University School of Medicine, Shanghai, China; ^3^Massachusetts General Hospital, Harvard Medical School, Boston, MA, United States; ^4^School of Management and Economics, Tianjin University, Tianjin, China; ^5^Surgical Planing Lab, Radiology Department, Brigham and Women's Hospital, Boston, MA, United States; ^6^School of Medicine, Tongji University, Shanghai, China

**Keywords:** facial recognition, deep learning, dynamic features, smiles, lip corners

For thousands of years of human history, we have learned how to fake or hide our genuine feelings and emotions to people around us intentionally or unconsciously. It is, indeed, an irony that this is what we view as emotional intelligence, and which we practice to win people over, display our politeness, tackle dilemmas, and deal with other complicated situations. Posed smiles are one of the most common faked expressions in our daily life. Indeed, it is a challenge for the computer vision system to recognize the genuine smile apart from posed smiles of an individual, and this may be difficult to interpret by humans too sometimes. Recently, an interesting work by Guo et al. (Guo et al., 2018) employed computer vision techniques to investigate the potential differences in the duration, intensity, speed, symmetry of the lip corners, and certain irregularities between genuine and posed smiles based on the UvA-NEMO Smile Database. The results are quite rewarding since they found that genuine smiles were correlated with higher onset, offset, apex, and total duration, as well as offset displacement and irregularity-b, compared with posed smiles. In addition, posed smiles were correlated with higher onset and offset speeds, irregularity-a, symmetry-a, and symmetry-d.

We cannot agree with the saying that only a handful of studies on the dynamic features of facial expressions have been conducted due to the lack of user-friendly analytic tools. On the contrary, in the past decades, hundreds of studies have focused on the dynamic features of facial expressions (Sandbach et al., 2012; Ko, 2018). Valstar et al. (2006) differentiated spontaneous brow actions from posed ones focusing on velocity, duration, and order of occurrence. Littlewort et al. (2009) distinguished fake pain from real pain by analyzing facial actions based on Gabor features. Dibeklioglu et al. (2012) analyzed the dynamics of eyelids, cheeks, and lip corners to tell genuine smiles from posed ones, and extracted 25 features, which were also cited by the author. Guo et al. (2018) said that not all these 25 features could be explained from a psychological perspective; hence, they extracted the duration, speed, intensity, symmetry, and irregularity aspects in their study. The question is why do all of the potential features need to be explained by psychological theory. It is possible that in this manner we may lose a lot of useful information to help distinguish genuine smiles from posed ones. Obviously, we still have great limited knowledge in psychology itself.

Indeed, the value of all the above-mentioned pioneering works should be appreciated, as they helped improve the recognition of posed smiles from spontaneous expressions over time. However, the hand-crafted features built by rules may lead to inadequate abstraction and representations. We are wondering whether the 25 features encompass the whole story to tell genuine smiles from posed ones, and how many of these extracted features would help the computer vision system to recognize posed smiles from genuine facial expressions. Obviously, there is still a lot of work left for us to consider and all of the features identified by different studies and extracted from different datasets need to be analyzed to help conduct the recognition performance. We cannot tell how much the dynamic features of the lip corners would help to differentiate genuine smiles from posed smiles from the diagnostic data presented in the current study.

Recently, deep learning has led to overwhelming performances in image or video processing over conventional methods such as facial recognition and classification (Peng et al., 2017; Rodriguez et al., 2017; Majumder et al., 2018; Yu et al., 2018). Many start-up companies have already built their businesses displaying outstanding performance in the field of facial recognition in security. It is not surprising that researchers have already adopted convolutional neural networking (CNN) to differentiate genuine smiles from posed ones, and the recognition performances have been promising (Kumar et al., 2017; Mandal et al., 2017). However, another question arises as to whether deep learning will take over this area and wipe out the necessity of studying hand-crafted features.

In reality the recognition performances, as consequences of deep learning in classifying the genuine smiles and posed smiles may rely heavily on the size of the training data. Unfortunately, datasets containing labeled genuine smiles and posed smiles are limited (Xu et al., 2017). However, the good news is that hand-crafted features combined with deep learning may have the potential to improve the recognition performances compared with deep learning alone supported by limited data (Pesteie et al., 2018). It is possible to build a hybrid model by inputting features from deep learning along with well-known features obtained from conventional methods into a classifier (Figure 1). We admit that deep learning has also been criticized for its level of interpretability, known as the black box. However, many researchers have realized the importance of solving the problem of the black box associated with deep learning, and solutions have been proposed to tackle the same (Gunning, 2017; Samek et al., 2017; Shwartz-Ziv and Tishby, 2017).

**Figure 1:**
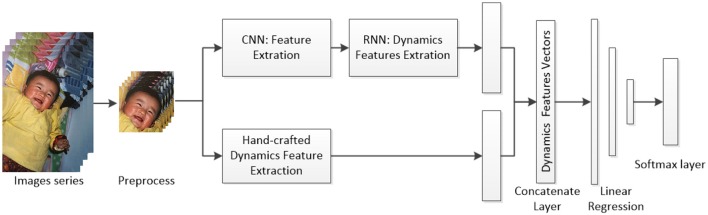
Hybrid model combining CNN and hand-crafted dynamic features (the smile picture belongs to the first author, and informed consent was obtained from the first author).

Considering outstanding recognition performance, we do believe that deep learning will dominate the area of image recognition and classification, including discriminating genuine smiles from posed ones. As for the black box, we should regard it as an accompanying aspect of deep learning, instead of being a mere limitation. It would be better if we can solve the problem of the black box similar to how Newton figured out why apples always fell to the ground. When that day comes, deep learning will have a greater impact than it has today, though we admit that more efforts are needed to solve the problem of the black box associated with deep learning.

## Author contributions

YL designed and wrote the manuscript. ZS revised the manuscript. HZ and LL gave critical comments. GF reviewed and approved the manuscript.

### Conflict of interest statement

The authors declare that the research was conducted in the absence of any commercial or financial relationships that could be construed as a potential conflict of interest.
